# Comparative evaluation of titanium miniplates versus 3D plates in mandibular fracture fixation: A clinical study

**DOI:** 10.6026/973206300222435

**Published:** 2026-04-30

**Authors:** M.L.V. Surya Mithra, Sandhya Joshi, Chaitanya Varangaonkar, Divija Jasti, Chiluveru Manoj Kumar, Lahari Priya Singareddy, Rahul Tiwari

**Affiliations:** 1 Department of Oral and Maxillofacial Surgery, Mamata Institute of Dental Sciences, Bachupally, Hyderabad, Telangana, India; 2 Department of Oral and Maxillofacial Surgery, National Academy of Medical Sciences, Bir Hospital, National Trauma Center, Kathmandu, Bagmati, Nepal; 3 Department of Oral and Maxillofacial Surgery, Shiyas and Ifthikar Medical Centre, Sharjah, United Arab Emirates; 4 Department of Dental Surgery, Mamata Dental College and Hospital, Khammam, Telangana, India; 5 Department of Oral and Maxillofacial Surgery, Sri Sai College of Dental Surgery, Vikarabad, Telangana, India; 6 Department of Public Health, St. Francis College, New York City, New York, USA; 7 Department of Dental Research Cell, Dr. D. Y. Pati Vidyapeeth College & Hospital, Dr. D. Y. Patil Vidyapeeth (Deemed to be University), Pimpri, Pune

**Keywords:** Mandibular fractures, titanium miniplates, three-dimensional plates, osteosynthesis, maxillofacial trauma

## Abstract

Inadequate fixation stability in mandibular fracture management often leads to postoperative complications and delayed functional recovery. Therefore, it is of interest to compare titanium miniplates and three-dimensional plates for mandibular fracture fixation in sixty randomized patients. Postoperative stability, infection, occlusion, pain and mouth opening were assessed over a twelve-week follow-up. 3D plate's showed superior stability with reduced operative time and lower complication rates. Thus, we show 3D plating systems provide more reliable fixation and faster functional rehabilitation than conventional miniplates.

## Background:

Mandibular fractures are among the most common facial skeletal injuries, representing a significant proportion of maxillofacial trauma cases worldwide [1]. They commonly result from road traffic accidents, interpersonal violence and sports injuries, requiring accurate reduction and stable fixation to restore occlusion and function [2]. Conventional titanium miniplates based on Champy's principles remain widely used due to adaptability and cost-effectiveness [3]. However, two-dimensional plate designs may provide insufficient resistance to torsional and bending forces, especially in angle and parasymphysis fractures [4]. Three-dimensional plating systems were developed with interconnected vertical and horizontal struts to improve load distribution and enhance fixation stability [5, 6]. Previous studies have reported improved postoperative bite force, reduced infection rates and better stability with 3D plates compared to conventional miniplates [7, 8]. However, variability in outcomes across fracture sites and clinical settings persists in the literature [9]. Therefore, it is of interest to comparative clinical evaluation of titanium miniplates and 3D plates remains relevant to determine optimal fixation strategies in mandibular fracture management.

## Materials and Methods:

This randomized, prospective clinical study included 60 patients aged 18-45 years presenting with isolated, non-comminuted mandibular fractures at a tertiary care hospital between January 2023 and March 2024.

## Patients were randomly allocated into two groups (n=30 each):

[1] Group I (Control): Treated with conventional 2.0 mm titanium miniplates.

[2] Group II (Study): Treated with 2.0 mm 3D titanium plates.

## Inclusion criteria:

Patients with symphysis, parasymphysis, or angle fractures suitable for open reduction and internal fixation (ORIF)

## Exclusion criteria:

Comminuted pathological fractures, systemic illness affecting bone healing or previous mandibular surgery. All patients underwent standard intraoral or transbuccal approaches under general anesthesia. Fixation was performed according to Champy's principles. Postoperative assessment included pain (VAS scale), swelling, infection, sensory deficit, occlusion, interincisal mouth opening and hardware failure at 1 week, 1 month and 3 months. Data were analyzed using SPSS v26.0. Chi-square and t-tests compared categorical and continuous variables. A p value <0.05 was considered statistically significant.

## Results:

Table 1 (see PDF) presents the comparative postoperative complications between titanium miniplates and 3D plates. Patients treated with 3D plates demonstrated a marked reduction in pain intensity, postoperative infection and hardware failure compared to those managed with conventional miniplates. Although paresthesia and malocclusion differences were not statistically significant, overall complication rates favored the 3D plate group, suggesting superior biomechanical stability and improved wound healing. These findings emphasize the clinical reliability of 3D plate fixation systems. Table 2 (see PDF) highlights the comparative evaluation of functional recovery between both fixation systems. Patients treated with 3D plates achieved greater interincisal mouth opening, stronger bite force and improved occlusal stability at all postoperative intervals. Enhanced early function at one week and sustained improvement up to twelve weeks reflect faster neuromuscular adaptation and stable fracture healing. These results affirm that 3D plates facilitate better masticatory rehabilitation and superior functional outcomes in mandibular fracture fixation.

## Discussion:

Rigid internal fixation aims to restore mandibular continuity, occlusal harmony and functional efficiency while minimizing postoperative morbidity [10]. Conventional titanium miniplates, although widely accepted, may inadequately counter complex torsional and bending stresses along the mandibular curvature [11]. Three-dimensional plate systems address these limitations by providing multidirectional stability and more uniform force distribution across the fracture site [12]. In the present study, lower infection rates, reduced hardware failure and improved occlusal stability were observed in the 3D plate group, indicating superior biomechanical performance. Similar findings have been reported by Sikora *et al.* who demonstrated better mouth opening and reduced sensory disturbances with 3D systems compared to miniplates [13]. Kaushik *et al.* also observed enhanced postoperative bite force and shorter operative time using curved locking strut plates [14]. Adhikari *et al.* reported reduced wound dehiscence and improved occlusal stability with three-dimensional fixation systems [15]. The box-like geometric configuration of 3D plates provides resistance to shear and torque forces, thereby reducing micro movement and promoting favourable bone healing [16, 17]. In addition, simplified plate adaptation contributes to reduced operative duration and improved surgical handling [18]. Limitations such as higher cost and adaptation difficulty near dental roots remain concerns, although recent preformed and patient-specific plate designs help overcome these issues [19, 20]. Overall, the present findings support the clinical reliability of three-dimensional plating systems as an effective alternative to conventional titanium miniplates for mandibular fracture fixation. This study provides prospective randomized clinical evidence comparing titanium miniplates and three-dimensional plates in mandibular fracture fixation. It demonstrates that 3D plates achieve superior postoperative stability and fewer complications. Data shows improved functional recovery with multidirectional fixation designs. Thus, adoption of 3D plating in maxillofacial trauma practice is recommended.

## Conclusion:

3D titanium plates provide superior fixation stability, reduced complication rates and faster functional recovery compared to conventional miniplates. They represent an effective alternative in mandibular fracture management. Future studies with larger cohorts and long-term follow-up are warranted to validate biomechanical and cost-effectiveness aspects.
